# Life after death: RIP1 and RIP3 move beyond necroptosis

**DOI:** 10.1038/cddiscovery.2016.56

**Published:** 2016-07-18

**Authors:** SB Berger, J Bertin, PJ Gough

**Affiliations:** 1 Host Defense Discovery Performance Unit, Infectious Disease Therapeutic Area, GlaxoSmithKline, Collegeville, PA 19426, USA; 2 Pattern Recognition Receptor Discovery Performance Unit, Immuno-inflammation Therapeutic Area, GlaxoSmithKline, Collegeville, PA 19426, USA

Over the last decade, programmed necrosis (or necroptosis) has been implicated as a significant driver of inflammation and pathology in both animal models^1^ and human disease,^2^ and as such, there has been a significant amount of interest in developing therapeutics to target this pathway. Within a couple of years after the characterization of necroptosis, receptor-interacting protein (RIP) 1 and RIP3 were identified as the two critical kinases responsible for mediating this form of cell death^3,4^ and more recent work has shown that the pseudokinase, mixed lineage kinase domain-like protein (MLKL), to be the direct executioner of programmed necrosis.^5^ Since these initial discoveries, RIP1, RIP3 and MLKL activation have all been synonymous with the onset of necroptosis. Despite some emerging literature describing additional roles for RIP1 and RIP3 kinase activity beyond triggering MLKL activation and necroptosis,^6,7^ it has largely been assumed by researchers that interventions targeting RIP1, RIP3 or MLKL were interchangeable. However, to this point, there have been no direct and comprehensive comparisons of these key mediators of necroptosis *in vivo*.

In a new paper published in *Cell Death and Differentiation*, for the first time Newton *et al.*^8^ have directly compared the individual contributions of RIP1, RIP3 and MLKL in numerous *in vivo* models of inflammation. In a tour de force effort using genetic inactivation of kinase activity or deletion of these proteins, the group compared head-to-head the role of RIP1, RIP3 and MLKL in 10 separate *in vivo* models. These models were selected based on previous experimental data using knockout mice or tool RIP1 inhibitors suggesting that necroptosis itself played a major contributing role to the observed pathologies. Through the studies in their paper, Newton *et al.*^8^ demonstrated that RIP1 kinase inactivation and RIP3 deficiency resulted in similar, significant protection from kidney ischemia reperfusion injury, myocardial infarction, A20 deficiency and high-dose TNF administration. Surprisingly, MLKL deficiency offered little to no protection in these models suggesting that RIP1/RIP3-dependent signaling, aside from necroptosis, was the primary driver of pathogenesis in these experimental models. Taken together, this work highlights that MLKL inhibition or inactivation is currently the only direct way to interrogate in isolation the role of necroptosis in driving inflammation and disease pathogenesis, as RIP1 and RIP3 are likely involved in additional biology beyond driving this form of cell death.

In addition to the aforementioned mouse models that were determined to be predominantly RIP1 and RIP3 dependent, the authors also identified other models including the dextran sodium sulfate-induced colitis and major cerebral artery occlusion stroke models, which were determined to be RIP1, RIP3 and MLKL independent. These results are quite surprising, as previous work has shown these systems to be RIP1 kinase-dependent using RIP1 tool inhibitor compounds such as necrostatin-1.^9,10^ Aside from inter-lab differences in animal housing and assay design, one plausible interpretation of these data is that off target effects, such as IDO inhibition, are largely responsible for driving the effects observed with these tool RIP1 inhibitor compounds. However, another potential explanation is that binding of these small molecules to the pocket of the RIP1 not only blocks kinase activity, but also results in a conformational change in the protein altering the scaffolding function of RIP1 and the ripoptosome. This hypothesis is supported by our recent unpublished data showing that the K45A RIP1 kinase dead mice are susceptible to high-dose TNF administration, while dosing a RIP1 inhibitor in these kinase dead mice results in complete protection from pathology. Additional work is now ongoing to better define these kinase activity-dependent *versus* scaffolding mechanisms of RIP1 inhibitors and to put them in the context of inflammation and disease.

In addition to the complexities around mechanistically understanding how RIP1 inhibitors are likely to exert their effects, it is now clear that our understanding of the biology of RIP1 and RIP3 beyond activating MLKL and necroptosis should be an area of significant focus. Recent work from various groups have now shown that in addition to driving necroptosis, RIP1 and RIP3 can also drive inflammasome assembly^11^ and RIP1, likely independent of RIP3, can function to directly initiate apoptosis and pro-inflammatory cytokine production.^6,12^ These results suggest that the necrosome may function as a decision node to functionally dictate the response to TNF and other stimuli. Further studies will be integral to understanding how the necrosome functions as a molecular switch in integrating an array of cellular signals and driving diverse cellular outcomes, which can contribute distinctly to disease pathogenesis.

With our evolving understanding of the role of RIP1, RIP3 and MLKL in contributing to pre-clinical models of inflammation and disease, it is imperative to better define the role of each of these proteins in driving human pathology. As such, it is vital to have the right tools that can be used in conjunction to identify pathway activation, including robust phospho-antibodies, which are starting to become commercially available and used on human disease tissue,^2^ potent and selective small-molecule inhibitors, which are now being generated and used in animal models of inflammation,^6^ and specific mutations in animal models, which can genetically and cleanly dissect out the contributions of these key signaling proteins.^8^ Based on the initial data from each of these tools, it is likely that therapeutics directed against targeting RIP1, RIP3 and MLKL will likely have very different impacts and liabilities in modulating the course of disease, with RIP1 inhibitors likely having the broadest therapeutic footprint (necroptosis, pathogenic forms of apoptosis, pro-inflammatory cytokine production and inflammasome assembly) based on the current body of pre-clinical data. However, under certain circumstances, MLKL inhibitors may also be desirable if a particular disease proves to be driven specifically by necroptosis. Additional work is now needed to bring together our growing understanding of RIP1, RIP3 and MLKL-specific biology with our growing repertoire of pathway-specific tools to lay the groundwork for the right intervention for the right clinical indication.
